# The Relationship of the Information Quantity of Urban Roadside Traffic Signs and Drivers’ Visibility Based on Information Transmission

**DOI:** 10.3390/ijerph182010976

**Published:** 2021-10-19

**Authors:** Kun Liu, Hongxing Deng

**Affiliations:** School of Traffic and Transportation, Northeast Forestry University, Harbin 150040, China; hhhtlkun@163.com

**Keywords:** urban roads, traffic signs, simulation, analysis, TSIQ, visibility

## Abstract

For the lack of quantitative basis of traffic sign combination information, this paper established a model of information quantity of urban road traffic signs by analyzing the driver’s information processing and the visual recognition of traffic signs combined with theories of informatics. It used various analytical methods to build a model of the relationship between the traffic sign information quantity (TSIQ) and the driver’s visual recognition. Based on factors, the relationship between the TSIQ and the driver’s visual recognition was studied and analyzed to provide a reference for the design of urban traffic sign layout information. The results show that the TSIQ can explain 61% of the driver’s recognition time (DRT). The more information the road traffic sign conveys, the longer DRT will be. The TSIQ’s threshold is 642 bits, and exceeding this value will cause information overload. Different influence factors have a certain impact on drivers’ visual recognition distance (VRD). The male VRD is shorter than the female. The VRD of the young driver is larger than the old driver. The VRD of a novice driver is longer than an experienced driver, while the visual recognition sign of an experienced driver is shorter.

## 1. Introduction

In recent years, with the prosperity of the economy and the development of the transportation industry, urbanization has been accelerated, road infrastructure has become better, people’s living standards have improved continuously, and their spiritual civilization has increased day by day. Traffic signs have increased gradually, that the department has set up different numbers and different forms of traffic signs on urban roads. However, as traffic signs continue to increase, their forms have become more complex and diverse. The consequence is that the driver’s visual burden increased virtually. Sometimes, the driver cannot understand the effective information correctly by the traffic sign at an appropriate time, resulting in the driver’s inability to change the vehicle’s state in time and miss the correct destination. Roadside traffic signs are the common forms of urban road traffic signs distributed on city roads. They play an important role in road traffic management and provide the information required for drivers to travel to the greatest extent [1]. The visibility of traffic signs directly affects their maximum effect. The amount of information carried by different traffic signs is different, and different amounts of information affect drivers’ visibility. Therefore, it is necessary to conduct in-depth research on the relationship between traffic sign information quantity and visibility.

Currently, many experts and scholars have carried out extensive research on traffic signs. They have achieved a series of fruitful research results. Johanssan et al. [2] explored the ability of different drivers to understand the layout of traffic signs from the perspective of human factors. Borteorte et al. [3] studied the relationship between the information conveyed by traffic signs and driving style. Through analysis, there were differences in the perception and processing of information by road users. Costa et al. [4] found that the driver’s gaze distance to the road sign was closer than the distance of visibility and used the ladder program to test and determine the time threshold that the accuracy of road sign recognition reached 75%. To make the traffic sign layout size more reasonable, Strawbridge [5] conducted a simulation experiment and obtained the reference value of the sign size according to the analysis result. Upchurch et al. [6] used driving simulation experiments to analyze and evaluate the effect of road signs on urban arterial roads and used them to revise MRTCD related specifications. Kaplan et al. [7] studied the relationship between drivers’ perception of road traffic safety, road sign information, observable and invisible factors. Myuncu et al. [8] studied the possibility of automatic identification of road traffic signs and conducted analysis experiments at different speeds. The results showed that the greater the speed, the longer the driver’s reaction time, the lower the traffic signs identified automatically. Huang et al. [9] took the driver’s factors as the research point and explored the significant relationship between driver comprehension and factors. The results showed that there were significant differences between male and female drivers in understanding road traffic signs. Different countries, education levels, and income levels will have an impact on driver understanding. Wogalter et al. [10] analyzed the psychological activities of drivers, selected social influence factors as independent variables, and explored the necessary factors for road sign design. Jamson et al. [11] explored the impact of bilingual traffic guidance signs on drivers’ attention under different lengths and complexity levels. They proposed that drivers read single-line, dual-line monolingual, and dual-line bilingual signs without affecting driving behavior. Fitzpatrick et al. [12] used driving simulation to compare and analyze the influence of different types of urban arterial road guidance signs on driving behavior. The results can provide theoretical guidance for the optimization of urban arterial road guidance signs. Schnell et al. [13] reduced the information collection time or improved the accuracy of information prompts. Larger and brighter signs can more effectively reduce the driver’s continuous visual recognition time and improve the accuracy of visual recognition, which greatly impacts road safety. Kim et al. [14] determined the letter size of traffic signs and the amount of layout information by analyzing factors such as the minimum vision sensitivity and reaction time required by drivers. Meeker et al. [15] studied the relationship between the character design of the sign layout and the size of the reference letter based on the highway complexity and information requirements and determined the information volume and combination arrangement of the layout design.

Pan et al. [16] took the external factors of the visibility of traffic signs as the research point. They used real-vehicle tests to explore the relationship between the visual distance of the signs and the speed and illumination. They found that the increase in vehicle speed will shorten the visible distance of the sign. In addition, the visual recognition effect of the sign at the same speed is forward light > night > backlight. The results showed that the information threshold of the traffic sign layout is seven pieces. Du et al. [17,18] used an eye tracker to conduct indoor simulation experiments to determine the reasonable number of road names for road signs and explored the quantitative relationship between traffic sign information and the visual recognition time. The study found that when the number of road names is 2 to 7, the driver’s visual recognition time does not exceed 2.5 s. The visual recognition time of target road name signs is significantly less than non-target roads. Jiang et al. [19] used experimental methods to study road signs, collected driver’s visual recognition data under different lighting conditions using eye trackers and GPS, built a parameter calculation model, and compared the setting parameters and the difference between normative values. The study found that, when visualizing the information of road signs, the driver’s gaze time will increase as the vehicle speed and the number of road names increase. The gaze time of the non-professional driver is longer than that of the professional driver, and the gaze time of the driver in the light is shorter than the gaze time at night. Zhao [20] studied the relationship between the average response time of the driver’s search for the target road name and the accuracy rate through a simulated driving test and found that the average response time of the driver is correlated positively with the amount of information on the traffic guidance sign layout. The response accuracy rate is correlated negatively with the amount of information. Jiang et al. [21] combined theoretical analysis and experimental results with exploring the quantitative relationship between the height of the signs and the visibility. The experimental results verify the rationality of the existing high standards of Chinese road signs and provide theoretical support for the scientific design of high road signs. Lv [22] combined the road network’s functional attributes and information nodes, proposed setting up road network traffic signs with different attribute categories and verified the effects of the new and old traffic signs through experiments. Rao et al. [23] defined the effective information for route search based on the traveler’s way-finding habits. They put forward a model for analyzing the guide signs’ information based on the planned route. The planned route guidance evaluated the conformity between the layout of the guide signs. Sun et al. [24] aimed at the problem that drivers often cannot distinguish the direction effectively when driving at a complex interchange and proposed a multi-color combination of road signs to verify the feasibility of the setting method through experiments. Wang [25] conducted an experimental study on the information volume of the guidance signs at the entrances and exits of the ring expressway, respectively explored a reasonable amount of information of guide signs under the cantilever and gantry type and gave the layout plan of the guide signs at the entrance and exit of the expressway around the city. Li et al. [26,27] compared Japan and China’s guide sign systems’ characteristics and analyzed the layout, location, content of guide signs at intersections and road sections. They also put forward relevant improvement suggestions, which guided the design and construction of road traffic signs, and provided a reference for revising the standard.

In summary, given the current research status, most of the research on road traffic signs by researchers is focused on the layout of traffic signs, the comprehension of signs, and the visibility of road signs, and a series of theoretical and practical results have been achieved [28,29,30,31,32,33,34,35,36,37,38,39,40,41,42,43]. However, the research is more on exploring single traffic signs and less on the combination of traffic signs. In particular, there are few types of research on the influence of traffic sign information and driver visibility in the combination of urban road traffic signs. In the past, most research on the amount of information focused on the number of road names. It often used the number of Chinese characters as indicators for research. The composition of the layout information of urban road traffic signs is complex. It is not comprehensive for using the number of road names and Chinese characters to measure sign information. The research focuses on textual information, and splitting the entire sign information volume cannot accurately distinguish the driver’s understanding of the information conveyed by the sign. Therefore, this article took urban road traffic signs as the object, explored the driver’s visual recognition process of traffic signs, and combined informatics theory for constructing a quantitative model of traffic signs information. It designed urban road traffic signs through scientific methods, recruited subjects to conduct indoor simulation experiments, and analyzed the results to obtain the quantitative relationship between TSIQ and visibility, based on considering factors, exploring the relationship between TSIQ and the driver’s VRD. The expected results can contribute to building a literature body of knowledge to help planners design traffic signs properly and reduce the frequency of behaviors such as detours and forced lane changes to improve driving comfort.

## 2. Theories and Models

### 2.1. The Driver’s Information Processing Process

**Figure 1:**
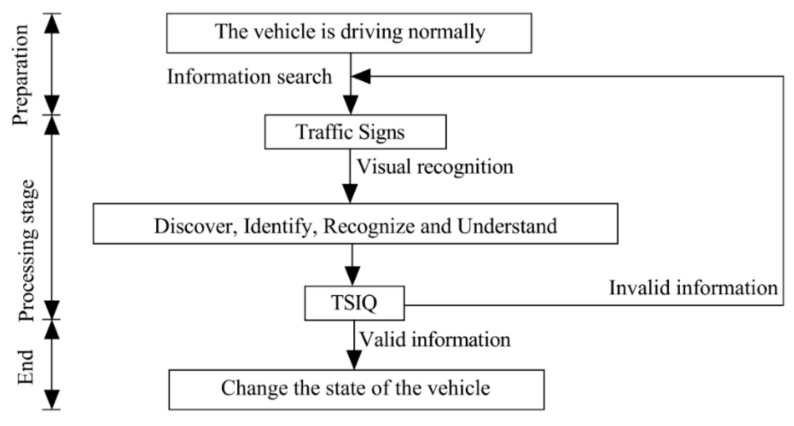
The driver’s information processing process.

When operating the vehicle on urban roads, the driver needs to recognize the traffic signs to obtain the required effective guidance to the greatest extent to manipulate the vehicles to reach their destinations. To this end, we need to understand the driver’s visual recognition process fully. In reality, the driver’s cognition and processing of traffic signs to convey information are very complicated. However, it usually simplifies into several major stages: discovery, recognition, understanding, and reaction. The driver’s information processing process is shown in Figure 1.

### 2.2. The Driver’s Visual Recognition Process

**Figure 2:**
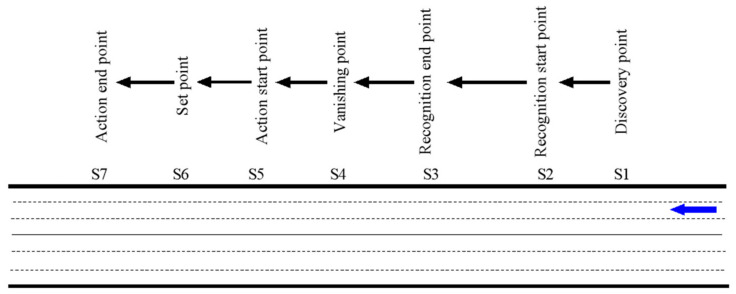
The driver’s visual recognition process.

Further research on the process of driver’s visual recognition of traffic signs can divide into the following stages: discovery of signs, identification of signs, reading of signs, understanding and decision-making, sign disappearance, the start of the action, and the end of the action. The detailed process of the driver’s visual recognition of traffic signs is shown in Figure 2.

When the driver operates the vehicle to reach point S1, the driver finds the existence of the traffic sign, but the driver cannot see the detailed information content of the traffic sign at this time. Continuing to drives normally to point S2, the driver can see the traffic sign information. Normally driving to point S3, the driver can see the traffic sign information starting to read the content of the guidance information that the sign can convey. When passing S3, the driver can understand the traffic signs and make his decisions. The driver takes action from S5 to change the travel status until the traffic signs disappear from the driver’s field of vision. Point S2 to point S6 is the driver’s VRD of the sign. The formula is:

(1) S=VT3.6+H2+L2tanθ,

where *V* is the driving speed of the vehicle, km/h; *T* is the visual recognition time of the driver, *s*; *H* is the distance difference between the driver’s line of sight and the traffic sign, *m*; *L* is the vertical distance between the driving direction of the vehicle and the sign, *m*; θ is the maximum viewing angle of the driver, usually θ = 15°.

### 2.3. The Traffic Sign Information Elements

**Figure 3:**
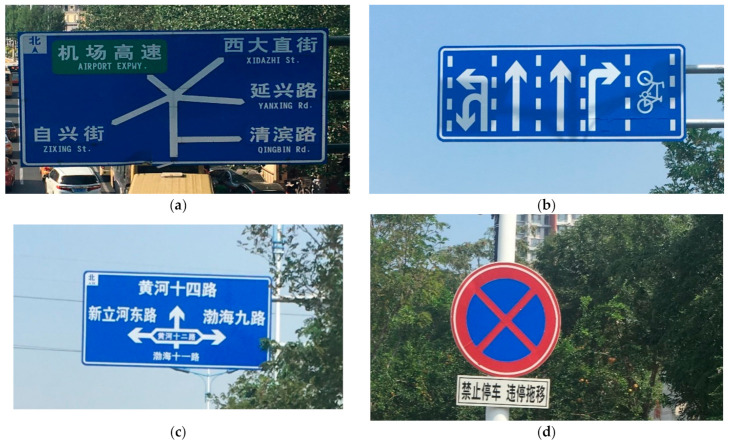
Examples of information elements contained in urban road traffic signs. (**a**) Traffic sign 1. (**b**) Traffic sign 2. (**c**) Traffic sign 3. (**d**) Traffic sign 4.

According to “Road Traffic Signs and Markings” [44] and “Code for Layout of Urban Road Traffic Signs and Markings” [45], the information types and information volume statistics in urban traffic signs are shown in Table 1, and the examples are shown in Figure 3 including:

- Chinese character: there are 3500 characters in total.
- English character: contains 26 English letters from A to Z.
- Arabic numeral: contains ten numbers from 0 to 9.
- Geometric figure: mainly includes six types, such as squares, circles, equilateral triangles, inverted equilateral triangles, octagons, and forks.
- Color: mainly consists of 11 kinds of color. Such as red, yellow, fluorescent yellow, blue, green, brown, black, white, orange, fluorescent orange, fluorescent yellow-green.
- Pointing symbol: it indicates different direction arrows or guidance symbols, about 30 kinds.
- Graphic or characteristic symbol: there are roughly 50 commonly used graphics and characteristic symbols in traffic signs.

### 2.4. The Traffic Sign Information Model

Information is a measurement of uncertainty degree and disorder in a certain situation or state. Its transmission process is a process from uncertainty to certainty. We can define traffic sign transmission information [46]: Road traffic sign transmission information describes the content of the attribute status or existence mode of each component of the traffic sign. The amount of information conveyed by a traffic sign is an indicator to evaluate the visibility and effectiveness of the traffic sign. Too much information conveyed by the sign will pressure the driver’s vision. On the contrary, the information content of the traffic sign can be recognized by the driver faster. Still, the side effect is that the information to be conveyed by the sign will not describe well, which increases the difficulty of the driver’s visual recognition and cannot play the role of the traffic sign.

American scholar Shannon first proposes the information theory. The amount of information carried by something is related to the probability of occurrence of content elements carried by the thing [47]. The amount of information is also called information entropy, and the unit is bits. Therefore, it assumed that the probability space of the transmission source of urban road traffic signs is (*X, Q, P*), and *Q* = (*x*_1_*, x*_2_*, …, x_n_*), and *P* = {*p*(*x*_1_)*, p*(*x*_2_)*, …, p*(*x_n_*)}, and the information entropy can be expressed as:

(2) $$I(X)=-\sum_{i=1}^{N}p(x_{i})\log_{a}^{p(x_{i})},$$

where *x_i_* is the *i*th event, *I* = 1, 2, …, *N*; *p*(*x_i_*) is the probability of the *i*th event information acting on the driver, and 0 ≤ *p*(*x_i_*) ≤ 1; *a* is the base of the logarithm, usually *a* = 2.

Therefore, we can derive the joint entropy and conditional entropy of traffic signs. Suppose that the *XY* probability space of the source is a joint probability space formed by (*X*, *Q_x_*, *P_x_*) and (*Y*, *Q_y_*, *P_y_*). From the above Formula (2), the joint entropy can be obtained as:

(3) $$I(X,Y)=-\sum_{i=1}^{N}\sum_{j=1}^{M}p(x_{i},y_{j})\log_{a}^{p(x_{i},y_{j})},$$

Thus, the conditional entropy of *X* under *Y* conditions by:

(4) $$I(X\left|Y\right.)=-\sum_{i=1}^{N}\sum_{j=1}^{M}p(y_{i})p(x_{i}\left|y_{j})\right.\log_{a}^{p(x_{i}\left|y_{j}\right.)},$$

where $p(y_{j})p(x_{i}\left|y_{j})=p(x_{i},y_{j})\right.$; therefore, Equation (4) can be written by:

(5) $$I(X\left|Y\right.)=-\sum_{i=1}^{N}\sum_{j=1}^{M}p(x_{i},y_{j})\log_{a}^{p(x_{i}\left|y_{j}\right.)},$$

Therefore, the information quantity for each traffic sign information element is obtained. The results are shown in Table 2.

## 3. Methods

### 3.1. The Experimental Design

In this paper, the experiment used a random sampling method to recruit 16 subjects with different genders, ages, and driving ages. It included eight male drivers, accounting for 50%. The subjects were required to have driving experience in different road environments, and their vision level was normal (correction vision 5.0 above). There was no color blindness.

**Figure 4:**
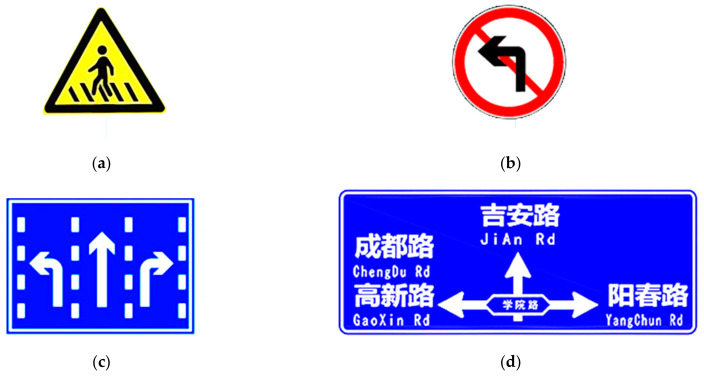
The sample design of the traffic sign layout in the experiment. (**a**) Warning sign. (**b**) Prohibition sign. (**c**) Indicator sign. (**d**) Guide sign.

**Figure 5:**
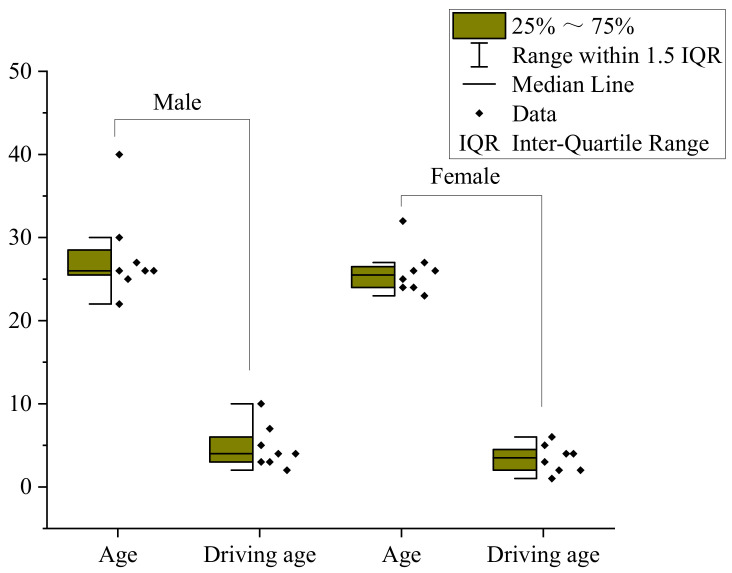
The subjects’ statistics.

The traffic sign layout designed by the experiment should reflect the difference in information quantity. The combination type conforms to the relevant standards [45] and conforms to the actual road setup type. The sample design of the traffic sign layout in the experiment is shown in Figure 4, and the subject’s information is shown in Figure 5.

### 3.2. The Experimental Process

**Figure 6:**
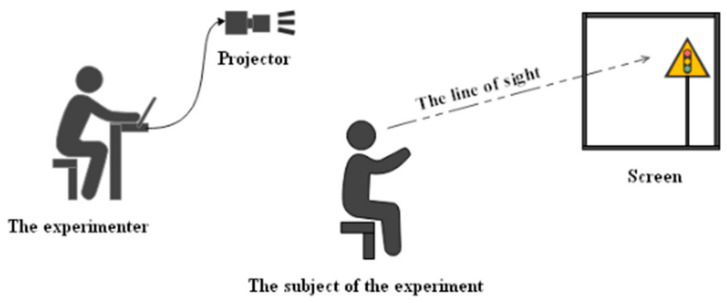
The concept diagram of the simulation experiment.

The paper used the indoor simulation experiment to explore the relationship between TSIQ and the driver’s visual recognition. The diagram of the simulation experiment is shown in Figure 6. Experimental hardware facilities involve computers, timers, projectors, and other software, including AutoCAD, Photoshop, etc. The experimental process is as follows:

1. Used a computer to load traffic sign picture information and a projector for a projection.
2. Experimenters informed the subject in advance of the experiment information.
3. When the subject found the target information, pressed the confirmation key, and answered quickly, the worker recorded the relevant information.
4. Cycled through the tests until all the subjects had completed the tests and finally summarized the data.

### 3.3. The Data Process

In the experiment, there is a “manual reaction time” in the process of traffic signs visual recognition. According to the relevant psychological theory [48], the value is 211 ms. Therefore, this value should be removed when processing experimental data. Furthermore, the experiment involves many sign pictures, and the error has large randomness, so it is necessary to conduct multiple experiments to minimize the error. 

**Figure 7:**
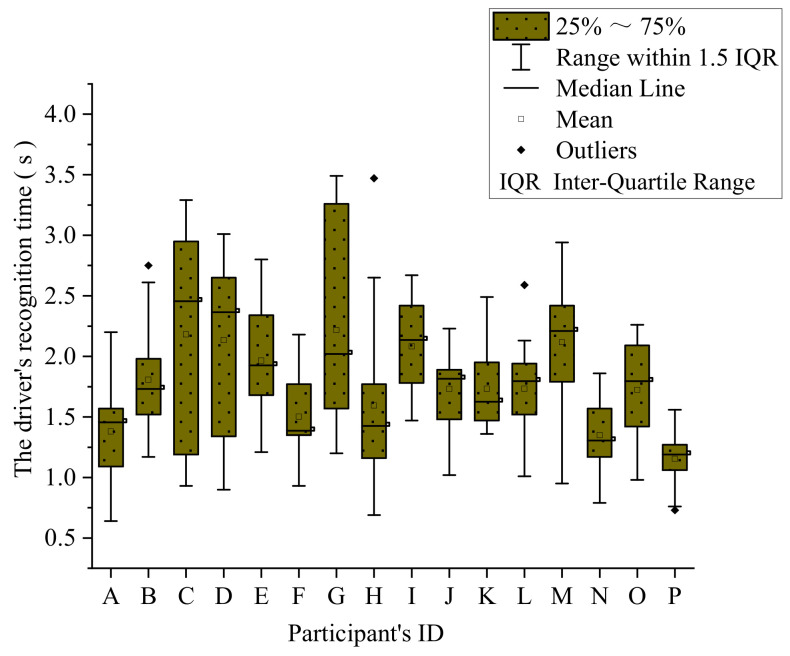
The box diagram of the data samples.

There were 18 groups of traffic signs in this experiment, and each group includes 16 subjects of the experiment. Therefore, a total of 288 data samples were collected. From the literature [49], generally, it is believed that the time for drivers to recognize and read traffic signs is between 0.5 to 2.0 s, but this experiment involves the combination of signs. It is necessary to increase the recognition time appropriately, and the article chooses 0.5 to 3.0 s. Part of the data exceeds the data because the subjects have physical problems such as distraction and nervousness during the experiment, which leads to errors in the operation. The original data box diagram is shown in Figure 7.

The data analysis shows that, in the original data, 10 data samples exceeded 3 s, and there were no data samples lower than 0.5 s. Therefore, the pass rate of the experimental data is 96.5%, reflecting the high efficiency of the data. From Figure 7, the maximum DRT was 3.490 s, the minimum DRT was 0.640 s, the mean DRT was 1.775 s, and the standard deviation was 0.586 s^2^. Thus, the DRT had not changed much. The statistical results are shown in Table 3.

**Figure 8:**
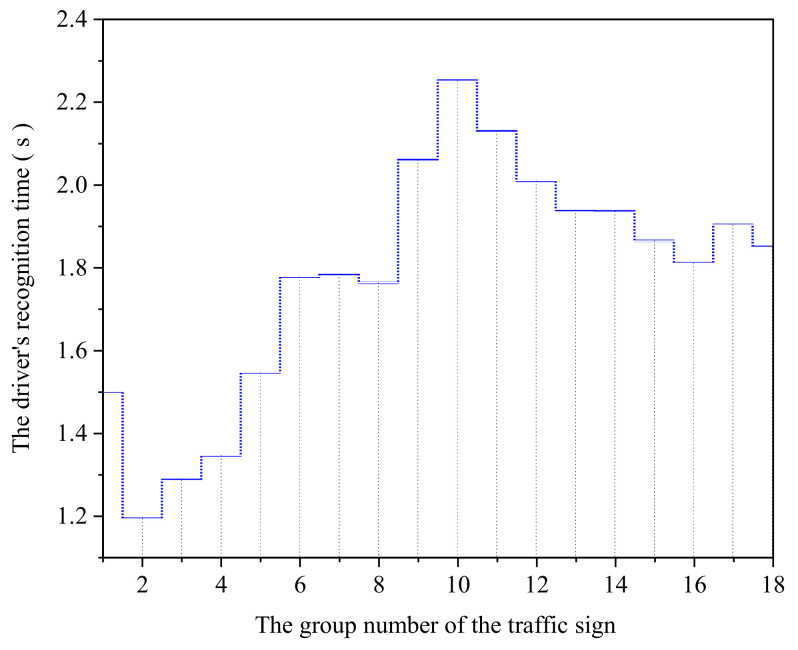
Modified driver’s recognition time.

The experiment was affected by artificial and random errors, which caused certain errors between the original and real data. Thus, to reduce the impact of errors, data need to be corrected. However, errors are inevitable in the experiment. Therefore, choose an appropriate estimation method to minimize the influence of the error caused by the unknown quantity estimation to obtain the best estimation under the normal mode [50]. Therefore, a robust estimation is adopted to process the data accordingly. The results are shown in Figure 8.

## 4. Results

### 4.1. The Relationship between TSIQ and DRT

Put samples into software for correlation analysis. There is a significant relationship between TSIQ and DRT at the confidence level (two-sided) of 0.01, and the correlation coefficient is 0.593. It reflects that there is a certain influence between the TSIQ and DRT. It is a positive influence. The more TSIQ, the longer DRT is required for the driver to recognize the traffic sign.

Thus, a regression model is used to explore the relationship between TSIQ and DRT to fit the experimental data, which involves linear, quadratic, cubic, logarithmic, exponential, power functions, etc. The model summary is shown in Table 4. 

**Figure 9:**
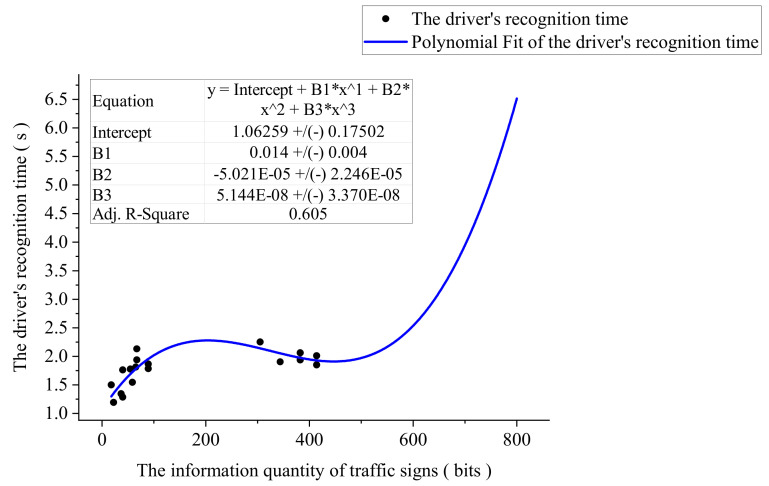
The visualization result of the model.

Table 4 shows that the significance level is less than 0.05. By comparing its goodness of fit value, the cubic function is the highest, the second is the quadratic function, and the lowest is the exponential function. The fitting effect of the cubic function regression model is the best, and its *R*^2^ = 0.674. Therefore, we used a cubic function to establish a model of the relationship between TSIQ and DRT. The visualization result of the model is shown in Figure 9. It shows that DRT increases with the increase of TSIQ.

Further analysis shows:

- When TSIQ is within the range of 0 to 447 bits, the curve increases smoothly, DRT does not change too much, and TSIQ is equal to 225 bits, DRT reaches its maximum value. When the TSIQ exceeds 225 bits, the DRT drops slightly. Analysis of the reasons shows that *R*^2^ = 0.605, indicating that the TSIQ can explain 61% of the change in DRT. Other factors may lead to changes in DRT, such as experimental conditions and the influence of human factors, which lead to a slight decrease in the curve.
- When TSIQ is greater than 447 bits, the curve is steeper, and DRT changes drastically. For example, TSIQ is 642 bits, DRT reaches 3 *s*. When it exceeds 642 bits, it will cause TSIQ to be overloaded, resulting in a significant increase in DRT and causing the driver not to recognize the traffic sign information accurately. Therefore, we can get a reasonable range of TSIQ, as shown in Table 5.

### 4.2. The Relationship between TSIQ and VRD

The driver’s visual recognition process knows that the DRT mainly includes recognition time, decision-making time, operation and reaction time, etc. From the literature [19], the decision-making time of the driver is 1.5 to 2.5 s, the article chooses 1.5 s, and the operation and reaction time of the driver is 1.0 to 2.0 s, with the article choosing 1.0 s. From the literature [51], the general design speed of urban arterial roads is 40 to 60 km/h. The width of the road is 3.5 m, the width of the non-motorized vehicle lane is 1 m, the height difference between the center of the sign and the driver’s line of sight is 1.3 m, the maximum field of view is 15°, and the distance between the sign and the edge of the road is about 0.25 m.

**Figure 10:**
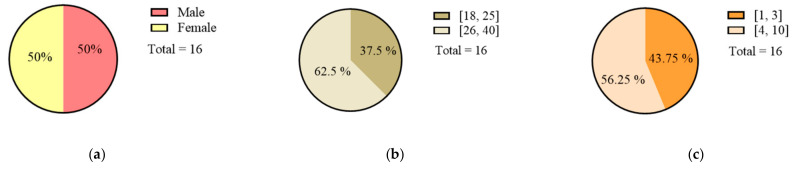
The descriptive statistics. (**a**) Genders. (**b**) Ages. (**c**) Driving ages.

To explore the relationship between TSIQ and VRD, with the help of analysis software, using statistical principles, starting from gender, age, driving experience, etc., to explore the relationship between TSIQ and VRD under the influence of different factors. We selected six groups of experimental samples whose sample parameters meet certain accuracy conditions. It contained two groups of data samples of different genders, ages, and driving experiences. The samples include eight male drivers, ten people over 25 years old, and nine people driving over three years. Detailed statistics are shown in Figure 10.

The correlation analysis shows that the correlation coefficient between the TSIQ and the male VRD is 0.472, and the correlation coefficient between the TSIQ and the female VRD is 0.616. The relationship coefficient between the TSIQ and the young VRD is 0.555, and the correlation coefficient of the old driver is 0.588. The correlation coefficient between the TSIQ and the VRD of the novice driver is 0.551, and the correlation coefficient with the experienced driver is 0.557. It can consider that the above relationships are significant when the confidence level is 0.05. That is, when the driver recognizes the traffic sign, the more information transmits, the longer the driver’s VRD. The correlation analysis table is shown in Table 6.

Through Table 7, the results show that the mean males VRD is smaller than females, reflecting that males have shorter VRD than females. The mean VRD of different ages tends to be equal, reflecting that the VRD of different ages has little difference. The reason may be that there is not much age difference in the samples selected in this experiment, and most are between 20 to 30 years old. The mean VRD of drivers who have been driving for more than three years is less than the mean VRD of drivers less than three years, reflecting that the VRD of novice drivers is longer than drivers with rich driving experience. Therefore, it concluded that VRD is 94 m approximately.

## 5. Conclusions

The paper analyzed the driver’s visual recognition of traffic signs on urban roads and used information theory to quantify the information transmitted by signs. Through indoor simulation experiments, combined with correlation analysis and regression analysis methods, explored the relationship between TSIQ and DRT and VRD, and it has given suggested values. The conclusions were obtained by research as follows:

There is a significant relationship between TSIQ and DRT. Through analysis, it shows that the TSIQ can explain 61% of the change in DRT. The greater TSIQ of the traffic sign, the longer DRT required by the driver, reflecting that the more information the sign conveys, the worse the driver’s visibility. Through analysis, it is known that the threshold of TSIQ is 642 bits. Therefore, TSIQ exceeds 642 bits, and the sign information will be overloaded, leading to a significant increase in DRT, failing to play the role of a traffic sign, and possibly causing serious consequences.

Drivers of different genders have different VRD. The mean male VRD is smaller than female, indicating that male drivers have shorter VRD, and males have better visibility than female drivers. Different ages have little difference in the mean VRD, and it reflects that the age difference will not have a big impact on the driver’s visibility. Through analysis, it shows that the mean VRD of young drivers is slightly higher than old drivers. Different driving ages will have an impact on VRD. Analysis shows that the VRD of novices is greater than experienced drivers, reflecting that the VRD required by novices is longer, and the visibility is lower than experienced drivers.

The research results of this paper have a good reference value for the information design of the traffic sign layout of urban roads, reducing the frequency of behaviors such as detours and forced lane changes to improve driving comfort. However, due to the influence of experimental conditions, sample size, and errors, it is necessary to refine the research results and make them more accurate and reference value.

## Figures and Tables

**Table 1:** The element statistics table.

The Serial Number	Type	N
1	Chinese character	3500
2	English character	26
3	Arabic numeral	10
4	Geometric figure	6
5	Color	11
6	Pointing symbol	30
7	Graphic or characteristic symbol	50

**Table 2:** The information quantity for each traffic sign information element.

The Serial Number	Type	Information Quantity/Bits
1	Chinese character	11.8
2	English character	4.7
3	Arabic numeral	3.3
4	Geometric figure	2.6
5	Color	3.5
6	Pointing symbol	4.9
7	Graphics or characteristic symbol	5.6

**Table 3:** The statistical description table of experimental data.

Type	N	Pass Rate	Minimum/s	Maximum/s	Mean/s	Std. Deviation/s^2^
DRT	288	96.5%	0.640	3.490	1.775	0.586

**Table 4:** The summary of the regression model.

Equation	Model Summary		
	R Square	F	Sig.
Linear	0.351	8.667	0.010
Logarithmic	0.520	17.335	0.001
Quadratic	0.620	12.249	0.001
Cubic	0.674	9.660	0.001
Power	0.511	16.741	0.001
Exponential	0.339	8.202	0.011

**Table 5:** The reasonable range of TSIQ.

Type	TSIQ’s Threshold Range/Bits	DRT’s Threshold Range/s
Excellent information	[0, 82]	[0, 1.9]
Suitable information	[82, 642]	[1.9, 3]
Information overload	[642, +∞)	[3, +∞)

**Table 6:** The correlation analysis table.

TSIQ			Genders		Ages		Driving Ages	
			Male	Female	[18, 25]	[26, 40]	[1, 3]	[4, 10]
		1						
**Genders**	**Male**	0.472 *	1					
	**Female**	0.616 **	0.755 **	1				
**Ages**	**[18, 25]**	0.555 *	0.759 **	0.927 **	1			
	**[26, 40]**	0.588 *	0.920 **	0.898 **	0.789 **	1		
**Driving ages**	**[1, 3]**	0.551 *	0.827 **	0.929 **	0.982 **	0.843 **	1	
	**[4, 10]**	0.557 **	0.873 **	0.867 **	0.736 **	0.978 **	0.769 **	1

* Correlation is significant at the 0.05 level (2-tailed). ** Correlation is significant at the 0.01 level (2-tailed).

**Table 7:** The VRD’s descriptive statistics.

Name		Type	Mean/m	Std. Deviation/m^2^
**VRD**	**Gender**	Male Female	93.371 95.512	3.516 7.233
	**Ages**	[18, 25] [26, 40]	94.281 94.219	5.471 4.896
	**Driving ages**	[1, 3] [4, 10]	96.834 92.356	5.811 4.327

## Data Availability

The data used to support the findings of this study are available from the corresponding author upon request.
